# In Experimental Tuberculosis Infection, the Bacteriostatic Function of Macrophages Is Activated by Th1 CD4^+^ T-Effectors in a Nitrite-Independent Manner

**DOI:** 10.3390/ijms26146573

**Published:** 2025-07-08

**Authors:** Vladimir V. Evstifeev, Konstantin B. Majorov, Vadim G. Avdienko, Vladimir V. Yeremeev, Galina S. Shepelkova

**Affiliations:** Central Tuberculosis Research Institute, Moscow 107564, Russia; vladimir-evstifeev@yandex.ru (V.V.E.); majorov@list.ru (K.B.M.);

**Keywords:** tuberculosis, macrophage, CD27, Th1 effector T cell, nitrite-independent mechanism of activation

## Abstract

The pivotal component in the protection against TB is the tissue macrophages (Mф). These cells have been demonstrated to play a crucial role in the elimination of pathogens and mycobacterial killing. Elucidation of the molecular and phenotypic events that determine the outcome of infection in Mф is fundamental to understanding the key features of these cells that are so important in fighting infection. Mф activation is driven by cytokines and other inflammatory mediators secreted by T lymphocytes. The interaction between *Mycobacterium tuberculosis* (Mtb) and host Мф has been the subject of extensive in vitro research. This dynamic interplay represents a pivotal step in the progression of mycobacterial infection because pulmonary macrophages constitute the primary line of defense against the pathogen, thereby serving as the initial immune cells to which Mtb must adapt to establish a replicative foothold within the host. Our studies have demonstrated that highly differentiated Th1 effectors with the CD27^low^ phenotype exhibit superior efficacy in activating both peritoneal (Mф: T cell ratio ranging from 125:1 to 625:1) and pulmonary macrophages (Mф: T cell ratio = 5:1) compared to cells with the CD27^high^ phenotype. Furthermore, our findings indicate that this activation mechanism is not contingent upon the production of reactive nitrogen species. To effectively activate the bacteriostatic function of macrophages, CD27^high^ T lymphocytes must differentiate into effectors with the CD27^low^ phenotype.

## 1. Introduction

As key components of the innate immune system, macrophages have an essential role in defending against pathogens through their phagocytic capabilities [1]. Macrophages are integral to the processes of maintaining tissue integrity, homeostasis, and wound healing, and regulating inflammation [2,3]. However, the efficacy of these agents is not assured, particularly in the context of combating *Mycobacterium tuberculosis* (Mtb), the primary causative agent of tuberculosis (TB). The respiratory tract is the main route of entry for Mtb into the host, with droplets or aerosols serving as the primary means of transmission. Following its traversal of the immune barrier of the respiratory tract, Mtb penetrates the lungs, where it comes into contact with macrophages. Macrophages capture Mtb by phagocytosis. They isolate the bacteria in phagosomes. Eventually, they destroy Mtb using a variety of mechanisms. However, mycobacteria have developed several ways to avoid being destroyed by macrophages. These ways have evolved as the mycobacteria have adapted to the host organism over time. The heterogeneity and plasticity of macrophages can be effectively exploited by Mtb for productive infection and dissemination. In order to prevail under immune or pharmacological pressures, the bacterium has the capacity to obtain and sustain a “metabolically retarded” latent form within macrophages [4,5,6]. Once inside macrophages, Mtb activates mechanisms that allow it to evade or resist the host immune response. Consequently, macrophages serve as suitable niches for Mtb survival, making it one of the most successful pathogens [5]. Consequently, following internalization by macrophages, Mtb can persist within the host for protracted periods of time. The ensuing struggle between the bacterium and the macrophage exerts a profound influence on the course and outcome of TB infection. In summary, macrophages play a dual role in both the regulation and progression of disease [7]. However, it is important to note that only activated macrophages possess the capacity to eliminate mycobacteria. The activation of infected macrophages is influenced by Th1 CD4^+^ effectors. It is widely accepted that IFNγ and TNF represent the principal cytokines involved in the activation of infected macrophages. However, the murine TB model demonstrated that the mycobactericidal capacity of pulmonary macrophages extracted from “naive” mice, devoid of prior exposure to mycobacteria, remains unaltered in the context of exogenous IFNγ stimulation [8].

In another mouse model of TB infection, it was demonstrated that effectors with a surface phenotype of CD4^+^CD44^high^CD62L^low^, infiltrating lung tissue during the active TB process, can be categorized into two subpopulations based on the presence of the CD27 receptor: CD27^high^ and CD27^low^. Furthermore, the CD27^low^ effector subpopulation has been demonstrated to exhibit increased production of inflammatory cytokines, including TNF and IFNγ, as well as the ability to activate infected macrophages of peritoneal exudate [9,10,11]. This study represents a logical progression from earlier research, providing compelling evidence that highly differentiated CD27^low^ effectors demonstrate superior efficacy in stimulating both peritoneal exudate macrophages and interstitial lung macrophages during in vitro experimental TB infection. The activation mechanism that has been identified is independent of NO production. In order to achieve optimal activation of the bacteriostatic function of macrophages, it is essential that CD27^high^ T-lymphocytes undergo further differentiation to effectors, adopting the CD27^low^ phenotype.

## 2. Results

### 2.1. Selective iNOS Inhibition Exerts No Effect on the Antimycobacterial Activity of Peritoneal Exudate Macrophages and Lung Interstitial Macrophages

**Figure 1:**
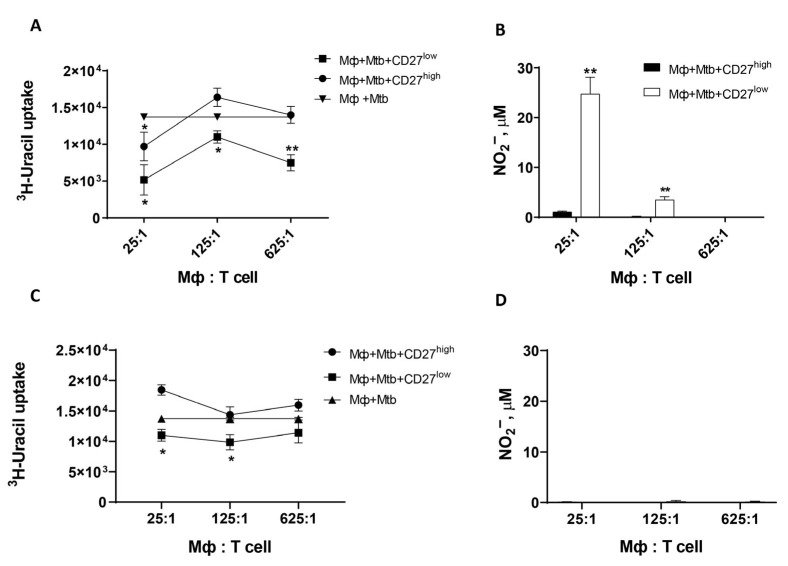
The initiation of the bacteriostatic function of Mtb-infected peritoneal exudate macrophages by highly differentiated CD27^low^ T lymphocytes is not contingent upon NO generation. Macrophages (Mф) from peritoneal exudate of B6 mouse underwent co-culture in vitro with Mtb (Mtb: macrophage = 5:1) and CD27^high^ (●, ■-columns) and CD27^low^ (■, □-columns) T lymphocytes in the presence or absence of L-NIL (**C**,**D**). After 72 h, the incorporation of 5,6-[^3^H]-uracil (**A**,**C**) and the amount of nitrite-anion (**B**,**D**) were measured in the cultures. (▲) shows suppression of Mtb growth by macrophages without additional stimulus. The figure shows data from one of three independent experiments with similar results (M ± SD, where n = 3 per group). *—significant difference from control (* *p* < 0.05; ** *p* < 0.001).

**Figure 2:**
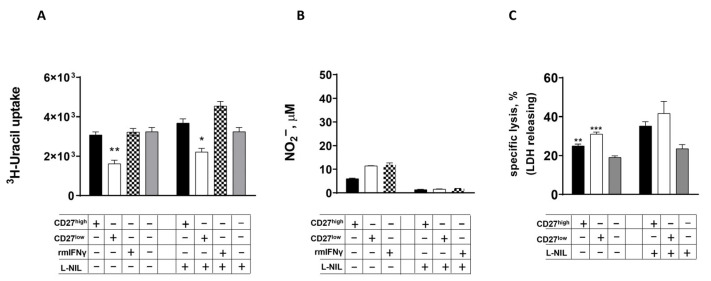
The antimycobacterial action of ‘naive’ interstitial lung macrophages activation by CD27^low^ T lymphocytes is not dependent on NO generation. ‘Naive’ interstitial Мф infected with Mtb (Mф: Mtb = 1:5) were cultured in vitro with Th-1 CD27^high^/CD27^low^ T lymphocytes, either with or without L-NIL (50 µM/well). Levels of MTB 5,6-[^3^H]-uracil incorporation (**A**), nitrite-anion concentration (**B**), and percentage of specific cell lysis (by LDH release) (**C**) were measured after 96 h in the cultures. Each bar in the figure denotes the presence of infected Mф (5 × 10^4^/well); the table below each figure indicates the presence of additional components within the system (“+”—present; “−”—absent). The figure shows data from one of two independent experiments with similar results (M ± SD, n = 3 per group). *—significant difference from control (* *p* < 0.05; ** *p* < 0.01; *** *p* < 0.001).

The objective of this study was to detect differences in the activation of the bacteriostatic function of macrophages by highly differentiated Th1 effectors. For this purpose, peritoneal exudate macrophages obtained from “naive” C57BL/6 (B6) mice were co-cultured with virulent Mtb (at a ratio of 5 Mtb per 1 macrophage). In the next step of the experiment, varying amounts of highly differentiated Th1 effectors (CD27^high^ or CD27^low^) were added to infected macrophages. The degree of activation of the bacteriostatic function of macrophages was determined by two methods. Firstly, the bacteriostatic function was judged by the suppression of Mtb growth in culture by the incorporation of 5,6-[^3^H]-uracil-labeled into the bacterium. Secondly, the bacteriostatic function was determined by the generation of NO^•^ by macrophages (as measured by the nitrite-anion concentration). In the context of this study, the positive control involved the addition of rmIFNγ to infected and “naïve” macrophages in wells. Macrophages, in the absence of additional stimuli, exhibited a moderate capacity to impede the proliferation of Mtb. Evidence for this was provided by a slight decrease in the incorporation of 5,6-[^3^H]-uracil Mtb (17,056 ± 415 imp/min in wells containing only mycobacteria; 13,731 ± 382 imp/min in wells with infected macrophages at *p* = 0.04). In this particular instance, there was a notable absence of activation of macrophages, as demonstrated by the absence of NO^•^ production (2 ± 0.4 μM) in the culture of infected macrophages. T cell titration experiments revealed that even single CD27^low^ effectors were capable of activating the bactericidal function of macrophages (at a T cell–macrophage ratio of 1:625), whereas CD27^high^ effectors only triggered this response at a ratio of 1 T lymphocyte per 25 macrophages (Figure 1A). Furthermore, a divergence in the capacity of these effectors to stimulate NO^•^ generation by infected macrophages was observed. A substantial variation in the stimulation of NO^•^ production was observed at T-cell–macrophage ratios of 1:25 and 1:125 (see Figure 1B). NO^•^ production was not registered in cultures with a T-cell–macrophage ratio of 1:625 (Figure 1B). The growth of Mtb was limited by interstitial macrophages in lung tissue obtained from “naïve” mice only when activated by T-lymphocytes with the CD27^low^ phenotype, at a 1:5 ratio (1 T-cell per 5 macrophages) in culture (Figure 2A). Macrophages were also activated at low concentrations of nitrite-anion in the supernatant (Figure 2B). This suggests that there is a way to make macrophages fight off TB that is not related to NO^•^.

The addition of the selective iNOS inhibitor L-NIL to a macrophage culture with Mtb resulted in the cessation of NO^•^ generation (Figure 1D and Figure 2B). However, unlike their CD27^high^ precursors, CD27^low^ T lymphocytes continued to activate the antimycobacterial activity of both peritoneal exudate macrophages (with T cell–macrophage ratios ranging from 1:25 to 1:125, as shown in Figure 1C) and “naive” lung interstitial macrophages (as shown in Figure 2A). These results confirmed the presence of a nitrite-independent mechanism that activates the antimycobacterial function of macrophages.

We quantified the cytotoxic effect of Mtb on macrophages using a biochemical method to determine the release of the macrophage cytosolic enzyme lactate dehydrogenase (LDH) into the extracellular medium. Figure 2C shows similar levels of eukaryotic cell lysis in cultures containing either CD27^high^ or CD27^low^. The nitrite-independent activation of “naive” pulmonary macrophages by CD27^low^ T-lymphocytes is confirmed by the lack of effect of L-NIL on the spontaneous lysis of eukaryotic cells (Figure 2C).

### 2.2. Macrophage Bacteriostatic Function Is Activated by CD70-CD27 Signaling

**Figure 3:**
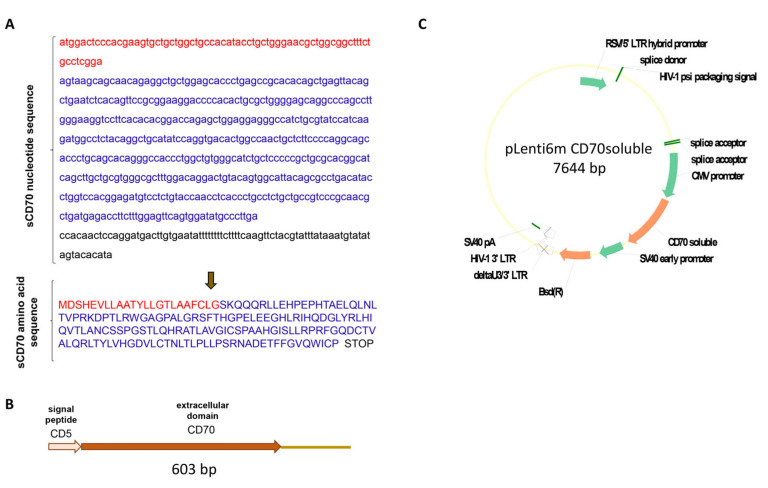
Cloning scheme for soluble CD70. (**A**) nucleotide and amino acid sequence of chimeric sCD70; (**B**,**C**) cloning scheme of the pLenti6mCD70soluble lentiviral construct containing the sequence of the artificial *cd70* gene. Red—CD5 protein signal peptide; blue—sCD70 protein.

Since our work demonstrated that CD27^low^ most effectively triggers the bacteriostatic ability of both peritoneal and pulmonary macrophages, and the differentiation of T effectors in TB goes in the direction of CD27^high^→CD27^low^ [12], it was suggested that the lower efficiency of T cells with the CD27^high^ phenotype in macrophage activation may be due to the fact that CD27^high^ effectors must be differentiated to CD27^low^. In order to put this hypothesis to the test, a soluble form of the ligand for the CD27 receptor, the activation molecule CD70, was cloned and produced. The chimeric soluble CD70 (sCD70) was produced by the extracellular domain of the CD70 protein (amino acids 41 to 195) being cloned together with the CD5 protein signal peptide (amino acids 1 to 23) into the lentiviral vector pLenti6 (Figure 3A–C).

**Figure 4:**
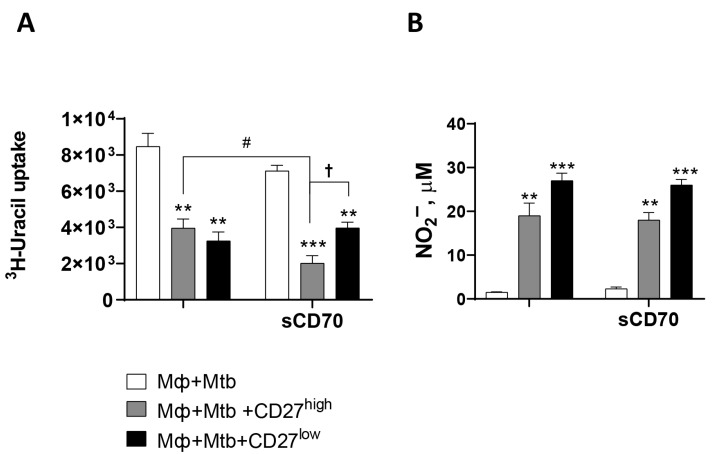
The effect of CD27^high^ → CD27^low^ effector differentiation on the activation of the bacteriostatic function of macrophages in experimental TB model in vitro. (**A**) Effect of sCD70 on suppression of Mtb growth by peritoneal exudate macrophages; (**B**) Effect of sCD70 on activation of bacteriostatic function of macrophages. Peritoneal exudate Мф from B6 mice were co-cultured with Mtb (Mф: Mtb = 1:5) and CD27^high^/CD27^low^ T lymphocytes (Mф: T lymphocyte = 1:25), either with or without sCD70 (12.5 ng/mL). The inclusion of 5,6-[^3^H]-uracil in the case of Mtb per se was 10,874 ± 657 imp/min. The figure shows data from one of three independent experiments with similar results (M ± SD, n = 3 per group). *—significant difference from Mф-infected controls (** *p* < 0.01; *** *p* < 0.001). #—significant difference between the T lymphocyte CD27^high^ groups (with and without sCD70). †—significant difference between groups with different effector T lymphocytes (*p* < 0.05).

The incorporation of sCD70 into the wells containing infected macrophages (devoid of T cells) did not exert any influence on mycobacterial growth restriction (Figure 4A) and, consequently, antimycobacterial activation of macrophages—NO^•^ production (Figure 4B). The addition of sCD70 to a culture of infected macrophages without T cells did not result in activation of the bacteriostatic function of phagocytes. There was not a big difference when sCD70 was added to a culture of infected macrophages with CD27^low^ T cells either (Figure 4). The soluble ligand, when added to the culture containing CD27^high^ T cells, led to an increased restriction of mycobacterial growth (Figure 4A), without affecting nitric oxide production by macrophages (Figure 4B).

## 3. Discussion

Given that Mtb is predominantly an intracellular parasite, the macrophage (most often the interstitial macrophage of lung tissue) is the primary cell in which a plethora of defense mechanisms are realized. Macrophages are among the first to come into contact with mycobacteria in TB infection. These cells are capable of eradicating mycobacteria, thereby facilitating the elimination of infection. At the same time, it is in these host cells that dormant Mtb can persist for decades. For mycobacterial growth to be effectively suppressed, macrophages must be activated. IFNγ and TNF, which are produced by CD4^+^ effector T lymphocytes, are believed to be the main cytokines responsible for macrophage activation at the site of infection in TB. Earlier studies demonstrated the existence of two subpopulations of highly differentiated Th1 effectors, CD44^high^CD62L^low^, which differ in their expression of CD27, a TNF receptor superfamily receptor [13]. A study of experimental TB in mice revealed that effector cells undergo differentiation directly in the infected tissue, specifically the lung, moving from a CD27^high^ to a CD27^low^ state [11]. The CD27^low^ T cell subpopulation is distinguished by increased secretion of proinflammatory cytokines, such as IFNγ and TNF [10]. These cytokines activate iNOS in macrophages, contributing to the production of reactive nitrogen species, particularly the NO^•^ radical [14,15]. High NO^•^ expression in response to cytokine or pathogen stimulation is an important component of the host’s defense against intracellular microorganisms [12]. In a mouse model of TB, NO^•^ plays a pivotal role in the eradication of *M. tuberculosis* by phagocytes [12].

Our work has shown that even a single, highly differentiated CD27^low^ lymphocyte (one T cell per 625 macrophages) can significantly suppress mycobacterial growth in peritoneal exudate macrophages (Figure 1A). These highly differentiated effectors can also activate the bacteriostatic function of “naive” interstitial lung macrophages (Figure 2A). These processes of macrophage activation are independent of NO^•^ radical generation by eukaryotic cells (Figure 1B and Figure 2B), which align with the results of studies by K. B. Mayorov and colleagues [8]. In that study, the addition of 100 U/mL of rmIFNγ to “naive” mouse pulmonary macrophages did not suppress mycobacterial growth or trigger the production of nitrite-anion by these cells in response to *M. tuberculosis* H37Rv stimulation. So, when we see that macrophages are getting more active and killing off the bacteria, and it does not seem to be because of nitrites, that tells us there might be a different way for the macrophages to get going.

Th1 CD4^+^ CD27^low^ effector T cells are an immunological biomarker of active tuberculosis and lung tissue destruction [10,16,17]. Decreased expression of the CD27 receptor leads to the formation of highly differentiated effector T lymphocytes that produce a wide range of cytokines and chemokines upon encountering an antigen. Significant increases in this subpopulation of T cells have been observed in the blood and at the site of infection (directly in lung tissue) in patients with active TB [16,17]. In a preceding investigation, we demonstrated that, in a murine model of experimental TB, effectors with a CD62L^low^CD27^high^ phenotype migrate to the site of infection (lung tissue), where they differentiate into effectors with a CD62L^low^CD27^low^ phenotype [10]. CD27 modulates the expansion of the repertoire of effector T cells when interacting with its ligand on antigen-presenting cells and lymphocytes, promoting T cell priming and survival [18]. The CD27 receptor’s ligand is the CD70 molecule, which is expressed on T and B lymphocytes, dendritic cells, and macrophages in both humans and mice [19]. CD27 and CD70 expression on the cell surface is inversely correlated, as CD27 involvement leads to a subsequent decrease in CD70 molecules [20]. CD70 is typically only expressed in the presence of a foreign agent, i.e., when an immune response is required [19,21]. The diminished efficacy of CD27^high^ T lymphocytes in stimulating macrophages in TB may be attributable to the necessity for CD27^high^ cells to undergo differentiation into CD27^low^ cells, as evidenced in our study (Figure 4A,B). The addition of recombinant soluble sCD70 to mixed cultures of Mtb-infected macrophages and CD4^+^ T lymphocytes with the CD27^high^ phenotype promoted enhanced suppression of Mtb growth in culture. To put it differently, for macrophages to effectively “kill” *M. tuberculosis*, CD27^high^ T effectors must differentiate into effectors with the CD27^low^ phenotype.

## 4. Materials and Methods

### 4.1. Animals and Mycobacterial Cultures

The experiment was conducted on female mice of the C57BL/6 (B6) lineage, ranging in age from two to four months, procured from the breeding facility of the Central Tuberculosis Research Institute (CTRI). All experimental procedures involving animals were approved by the Local Ethics Committee of CTRI (protocol #6, 14 January 2013 and protocol #1/2, 17 January 2025). Induction of experimental TB in mice was performed by intravenous injection of Mtb (strain H37Rv (Pasteur) from the collection of CTRI at a dose of 10^5^ CFU/mouse.

### 4.2. Procuring Macrophage Suspension

The macrophages of the peritoneal exudate were obtained using the method previously described by Shepelkova et al. [11]. Macrophage culture enrichment was achieved by adhering cells to Petri dishes for tissue culture. Lung cell suspension was obtained using the modified method of Holt et al. [22,23]. To obtain pulmonary interstitial macrophages, the lung cell suspension was first filtered to separate the cells, which were then seeded onto Petri dishes for eukaryotic cultures. Non-adherent cells were removed. The percentage of nonspecific esterase-positive cells was greater than 85%.

### 4.3. CD4^+^ T Lymphocyte Isolation

Highly differentiated CD4^+^CD62L^low^CD27^high^ and CD4^+^CD62L^low^CD27^high^ T lymphocytes were obtained by magnetic field sorting of splenocytes from intravenously infected mice 21 days after infection using monoclonal, PE/biotin-labeled antibodies (CD62L-biotin and CD27-PE, (Becton, Dikinson and Company BD Biosciences, San Jose, CA, USA), as well as a CD4^+^ T Cell Isolation Kit, Anti-PE MicroBeads and Anti-biotin MicroBeads (Miltenyi Biotec, Bergisch Gladbach, Germany), according to the manufacturers’ recommendations.

### 4.4. Mycobacteria Viability Determination

The viability of mycobacteria in cultures was assessed by the bacteria’s uptake of 5,6-[^3^H]-uracil [11,24]. Additionally, 1 μCi/wells of 5,6-[^3^H]-uracil (Isotop, St. Petersburg, Russia) was added for the last 18 h.

### 4.5. Determination of Nitric Oxide (NO^•^) Production

The production of reactive nitrogen species by macrophages was evaluated by measuring the nitrite-anion concentration in culture supernatants using the Griess reagent [8]. The inhibitor N6-(1-iminoethyl)-L-lysine (L-NIL) (Sigma-Aldrich, St. Louis, MO, USA) at a dose of 0.5 mM was used to selectively block inducible NO synthase (iNOS). In some experiments with mouse macrophages, 100 U/well of recombinant murine IFNγ (rmIFNγ) (MERCK, Sigma Aldrich, Darmstadt, Germany) was added to the culture.

### 4.6. LDH Release from Macrophages

The degree of macrophage lysis was determined by measuring the enzymatic activity of lactate dehydrogenase (LDH) in culture supernatants using a CytoTox 96 kit (Promega, Madison, WI, USA) according to the manufacturer’s recommendations. The percentage of specific lysis was calculated using the following formula: (A_490_ in experimental wells − A_490_ after spontaneous release)/(A_490_ after total lysis − A_490_ after spontaneous release) × 100.

### 4.7. DNA and RNA Isolation

Genomic DNA was isolated from the tail of a B6 mouse using a Genomic DNA Purification Kit (Promega, Madison, WI, USA), following the manufacturer’s instructions. Total RNA was isolated from mouse lung tissue using miRNeasy Tissue/Cells Advanced Kits (QIAGEN, Gmbh, Hilden, Germany) according to the manufacturer’s recommendations without additional modifications. cDNA was obtained using a SuperScript™ III Reverse Transcriptase kit (ThermoFisher Scientific, Waltham, MA, USA).

### 4.8. Preparation of Chimeric Soluble CD70

The chimeric sCD70 protein was produced through the fusion of the CD5 protein signal peptide (amino acids 1–23) and the extracellular domain of the CD70 protein (amino acids 41–195). The nucleotide sequence encoding the CD5 protein signal peptide was obtained by PCR using the following primers:

- CD5speF:

5′-TTATTAACTAGTCCGATGTGGTGCTGGC-3′

- CD5bspmR:

5′TTATTAGCAGGTATGTGGCAGCCAGCAGCAGCACT3′.

The genomic DNA of the B6 mice was used as the matrix for the CD5 protein signal peptide. The nucleotide sequence encoding the extracellular fragment of the CD70 protein gene was obtained from the lung tissue cDNA of “naive” B6 mice using the following primers:

- CD70bspmF:

5′TTATTAACCTGCTGCTGGGAACGCTGGCGGCTTTCTGCCTGCCTAGGAAGTAAGCAGCAGCAACAGAGGGCTGCTGGAGCACCCTGA3′

- CD70ecor5R:

5′TAATAAGATATATATCTATGTGTGTACTATATATATACATTTATAAATACGTGAA 3′.

The obtained fragments were hydrolyzed using the following restriction endonucleases: Spe I/BspM I (CD5) and BspM I/EcoR V (CD70). These were then cloned into the pLenti6m vector, which was hydrolyzed using Spe I/EcoR V. All restriction endonucleases were obtained from New England Biolabs (Ipswich, MA, USA).

### 4.9. Production of Stocks Containing Lentiviral Particles

Lentiviral stocks were produced in HEK293T cells. A Lipofectamine-DNA complex was prepared according to the manufacturer’s recommendations (Invitrogen, ThermoFisher Scientific, Waltham, MA, USA). The plasmids pLenti6mCD70soluble (with the soluble form of CD70) and the packaging plasmids (pJS86 and pCMVdR8.2) were introduced at a ratio of 1:3:4, respectively. At 12 h and 24 h intervals, stocks containing lentiviral particles were collected. Each stock was concentrated by ultracentrifugation; the supernatant was filtered through a 0.45 μm pore diameter filter, and the stock was stored at −80 °C.

### 4.10. Lentiviral Transduction of Cells

This process was performed in mouse embryonic fibroblasts. Lentiviral particles containing the green fluorescent protein (GFP) gene were used to evaluate transduction efficiency. After visualizing the GFP-positive cells, the cells were selected using a medium containing blasticidin (1 μg/mL) (InvivoGen, San Diego, CA, USA). Non-transduced fibroblasts were used as a control.

The concentration of recombinant mouse CD70 (sCD70) was determined using an enzyme-linked immunosorbent assay with a Mouse CD70 Antigen/TNFSF7 (CD70) ELISA Kit (Abbexa Ltd., Cambridge, UK).

### 4.11. Statistical Analysis

Statistical analysis was performed using a multiple *t*-test with Holm–Sidak multiple correction (Hoim-Sidak method) in GraphPad Prism 8.4.3 (GraphPad Software, San Diego, CA, USA).

## Data Availability

The original contributions presented in this study are included in the article. Further inquiries can be directed to the corresponding authors (V.V.Y. and S.G.S.)

## Abbreviations

The following abbreviations are used in this manuscript:

B6	C57BL/6 mice
CTRI	Central Tuberculosis Research Institute
imp/min	Impulse per minute
LD	Linear dichroism
iNOS	inducible NO synthase
LDH	lactate dehydrogenase
L-NIL	N6-(1-iminoethyl)-L-lysine
Mtb	*Mycobacterium tuberculosis*
Мф	macrophages
sCD70	soluble mouse CD70
TB	tuberculosis
